# Editorial: Modulation of neural circuits and plasticity underlying opioid analgesia, tolerance, and addiction

**DOI:** 10.3389/fnins.2023.1191030

**Published:** 2023-04-04

**Authors:** Wei Jiang, Wangyuan Zou, Zengyou Ye

**Affiliations:** ^1^Shanghai Sixth People's Hospital, Shanghai Jiao Tong University, Shanghai, China; ^2^Department of Anesthesiology, Xiangya Hospital, Central South University, Changsha, China; ^3^National Institute of Dental and Craniofacial Research, National Institutes of Health, Bethesda, MD, United States

**Keywords:** chronic opioid use, pain management, microglia, kappa opioid receptor, naloxone, hyperalgesia, circular RNA, post-herpetic neuralgia

The modulation of neural circuits and plasticity underlying opioid effects, including analgesia, tolerance, and addiction, is a critical research area. Chronic opioid use can lead to changes in neural circuitry, pain processing, reward, and motivation, leading to tolerance and addiction. Therefore, understanding the neural mechanisms underlying these processes is vital for developing new treatments for pain management and opioid addiction.

Liu-Chen and Huang reviewed the potential therapeutic benefits of kappa opioid receptor (KOR) agonists, such as analgesia and treatment of substance use and demyelinating diseases, while minimizing their adverse side effects. Xing et al. investigated the role of circular RNAs (circRNAs) and N6-methyladenosine (m6A) modifications of RNA in morphine tolerance. Meanwhile, McKendrick et al. investigated the role of the anterior cingulate cortex (ACC) in context-induced opioid seeking behavior in mice.

Antoine et al. explored the potential relationship between opioid use disorder (OUD) and microglia, the resident macrophages of the central nervous system (CNS). Furthermore, they suggested that the inflammatory response mediated by the microglia could contribute to the pathophysiology of SUDs, particularly OUD.

Qian et al. conducted a clinical trial investigating the efficacy of low-dose naloxone in reducing the incidence of sufentanil-induced cough (SIC) and postoperative nausea and vomiting (PONV). Additionally, Yiping et al. conducted a randomized controlled study to evaluate the safety and effectiveness of epidural morphine or hydromorphone in patients with post-herpetic neuralgia (PHN).

Finally, Ma et al. proposed a hypothesis that comprehensive pain assessment using scientific and technological means, combined with psychological assessment pictures and existing scales, may provide a new method for more effective clinical treatment of pain, especially chronic severe pain. Additionally, Jia et al. investigated whether nalmefene, dexmedetomidine, and both drugs combined prevent remifentanil-induced hyperalgesia in patients undergoing elective laparoscopic gynecological surgery.

In summary, these reviews and studies shed light on the neural mechanisms underlying the effects of opioids, and the potential therapeutic benefits, while minimizing their adverse side effects. The updates included in this Research Topic are essential for developing new treatments for pain management and opioid addiction. They would be useful references for all readers conducting opioid research.

## Author contributions

All authors listed have made a substantial, direct, and intellectual contribution to the work and approved it for publication.

